# A Method for Neutralizing Entropy Measurement-Based Ransomware Detection Technologies Using Encoding Algorithms

**DOI:** 10.3390/e24020239

**Published:** 2022-02-04

**Authors:** Jaehyuk Lee, Kyungroul Lee

**Affiliations:** School of Computer Software, Daegu Catholic University, Gyeongsan 38430, Korea; gurtmggg@cu.ac.kr

**Keywords:** ransomware, encoding algorithms, entropy measurement, malicious code

## Abstract

Ransomware consists of malicious codes that restrict users from accessing their own files while demanding a ransom payment. Since the advent of ransomware, new and variant ransomwares have caused critical damage around the world, thus prompting the study of detection and prevention technologies against ransomware. Ransomware encrypts files, and encrypted files have a characteristic of increasing entropy. Due to this characteristic, a defense technology has emerged for detecting ransomware-infected files by measuring the entropy of clean and encrypted files based on a derived entropy threshold. Accordingly, attackers have applied a method in which entropy does not increase even if the files are encrypted, such that the ransomware-infected files cannot be detected through changes in entropy. Therefore, if the attacker applies a base64 encoding algorithm to the encrypted files, files infected by ransomware will have a low entropy value. This can eventually neutralize the technology for detecting files infected from ransomware based on entropy measurement. Therefore, in this paper, we propose a method to neutralize ransomware detection technologies using a more sophisticated entropy measurement method by applying various encoding algorithms including base64 and various file formats. To this end, we analyze the limitations and problems of the existing entropy measurement-based ransomware detection technologies using the encoding algorithm, and we propose a more effective neutralization method of ransomware detection technologies based on the analysis results.

## 1. Introduction

Ransomware consists of malicious codes that restrict users’ access to their own files by encrypting them and then demanding a ransom in exchange for decrypting them; the first instance was written by Joseph Popp in 1989 [1]. According to the latest ransomware trends analysis of the Korea Internet and Security Agency (KISA), there were 22 reports in South Korea in 2018, 39 cases in 2019, 127 cases in 2020, and 97 cases in 2021 as of July, meaning that this trend has shown a steady increase over the past three years [2,3]. This ransomware trend is causing critical damage not only in South Korea, but also worldwide [2,3]. Therefore, various methods for preventing and detecting ransomware infection have been studied, and these methods can be classified into file-based detection, system-based detection, and resource-based behavior detection [4,5,6,7].

The file-based detection method detects ransomware based on signatures that have malicious actions in a specific file format. However, this method does not detect new and variant ransomware [8]. The system-based detection method is a method of detecting malicious behavior through integrity verification and the blocking of malicious behavior. However, this detection method has problems, including a false-positive detection ratio and requiring a long time for integrity verification [9]. The resource-based behavior detection method detects ransomware based on increased CPU usage and I/O throughput while the ransomware accesses and encrypts files. However, this detection method also requires a lot of time to collect CPU and I/O resource information, and it has a high false-positive ratio [9].

Due to the problems with the above ransomware detection methods, a recent work examined methods for detecting ransomware-infected files after the victim’s files have been encrypted, rather than preventing or detecting ransomware. Nevertheless, it was difficult to detect if a binary encoding algorithm such as base64 had been applied, because this detection method detects ransomware-infected files based on the characteristic of increasing entropy during file encryption [10]. As a result, it was possible to neutralize the method for detecting ransomware-infected files. Nonetheless, in this study, there was also a difference in entropy between clean files and files to which the encoding algorithm is applied in specific file formats. Thus, if this entropy difference is applied as an entropy threshold, then ransomware-infected files can be detected. In addition, in that prior work, only base64 was applied as an encoding algorithm; experiments with various file formats were not applied; and lastly, the attacker’s point of view regarding the neutralization of the detection method was not considered.

Therefore, this study was conducted with the aim of deriving an encoding algorithm with the best effect from the attacker’s point of view in order to neutralize the entropy-based ransomware detection method, as well as devising a way to neutralize the detection method of a defender so that it is impossible to detect ransomware using file entropy measurement methods. We expect that the results derived in this work will be helpful for future research aiming towards the development of entropy-based ransomware detection technology with optimal performance.

The contributions of this paper are as follows.

- In this work, to propose a method for neutralizing ransomware detection from the attacker’s point of view, and to derive a neutralization method with the highest efficiency, ransomware defense technology or the defender’s position was considered.
- The existing methods for neutralizing ransomware detection only apply a base64 encoding algorithm to decrease the entropy of files infected from ransomware. Nevertheless, even if the base64 encoding algorithm is applied, there is still a difference in entropy between plaintext and ciphertext, so it is possible to detect ransomware-infected files by measuring entropy. Moreover, a specific file format has a characteristic in that the entropy of the plaintext is decreased significantly, thus allowing ransomware-infected files to be detected based on abnormal measurements. Therefore, in this study, we derived the most effective neutralization method from the attacker’s point of view by using various encoding algorithms other than the base64 encoding algorithm.
- The proposed method derives entropy thresholds for each file format to detect ransomware-infected files in various file formats. Through this process, we determined the best neutralization methods for each file format and each encoding algorithm.
- To neutralize the method of detecting files infected with ransomware based on entropy measurement, this study derived an optimal encoding method in which the entropy of plaintext files and encoded files are almost the same. Finally, a ransomware detection neutralization method using the derived encoding method was developed, and is proposed in this paper. Therefore, by simply measuring the entropy of a file, a defender can cause confusion in the detection of files infected with ransomware, and a defender cannot effectively detect it.

## 2. Prior Knowledge and Related Works

To detect ransomware and to prevent any associated harm, various detection methods have been studied. To detect ransomware, methods such as file-based detection, system-based behavior detection, and resource-based behavior detection have been proposed. However, these methods have obvious drawbacks and limitations. For this reason, methods for detecting and identifying ransomware-infected files using the measured value of entropy of the files on the user’s system have mainly been studied in recent years. Therefore, this section describes ransomware detection in a user system as well as prior knowledge and related works for entropy-based ransomware detection technology. Prior knowledge explains information entropy, which is information for determining whether ransomware has infected any files in the user system, and binary-text methods for neutralization measures. Previous studies have demonstrated entropy measurement-based ransomware detection technology using the characteristics of encryption algorithms and the neutralization of entropy measurement-based ransomware detection technology by applying the base64 encoding method.

### 2.1. Prior Knowledge

#### 2.1.1. Information Entropy

Entropy is a term that indicates the degree of unpredictability—namely, the amount of uncertainty—and information entropy is a concept that is used in computer science. Information entropy was first proposed by Shannon and refers to the expected value of the amount of information. In general, eight bits have a value from 0 to 8 [11,12]. Here, the closer the value is to zero, the lower the entropy, which means that the data are non-uniform. Conversely, the closer to eight, the higher the entropy, which means that the data are uniform. To calculate information entropy, the formula for calculating entropy based on file data is shown below. *H*(*x*) is information entropy and *p*(*i*) is the probability that is generated [9].

(1) $$H\left(x\right)=-\overset{n}{\underset{i=1}{\sum }}p\left(i\right)log_{2}p\left(i\right)$$

#### 2.1.2. Binary-Text Encoding Algorithm

**Figure 1:**
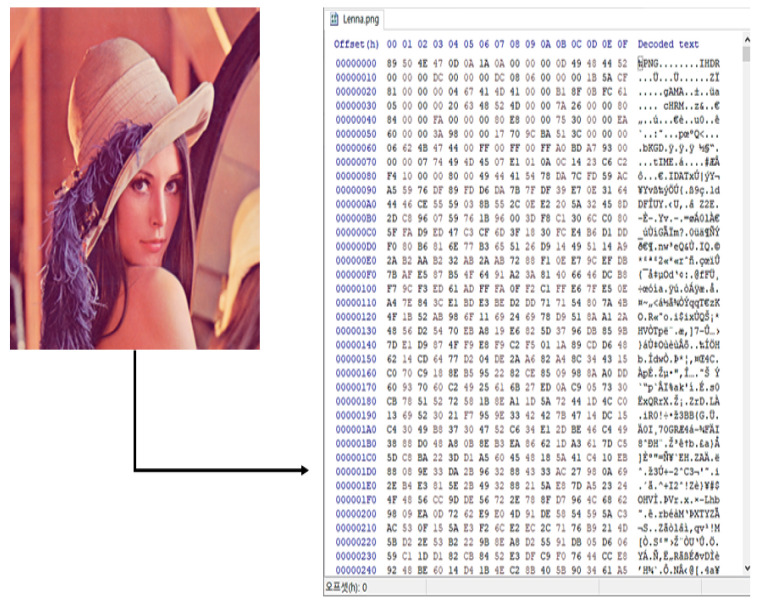
An example of a picture file, which is one of the binary file formats.

Early encoding algorithms appeared to transmit files consisting of ASCII (American Standard Code for Information Interchange) characters on the network. At this time, for the data exchange system to transfer a file composed of binary data, it was necessary to encode the data into ASCII characters, and then decoding was used to recover the received ASCII characters into binary data. Here, a binary file is a file composed of 0’s and 1’s for the purpose of file storage and information processing on a computer, and it is different from the txt or csv file format, each of which is a text file type [10]. Figure 1 shows an example of a picture file, which is one of the binary file formats.

As shown in the figure above, when the original image file on the left is opened with a hexadecimal editor, a value between 0×00 and 0×FF appears, as shown on the right. All file formats in the computer are expressed in binary, so it is difficult for a human, as opposed to a computer, to intuitively recognize data. For this reason, binary-text encoding algorithms for converting binary data into text data that can be read by humans have emerged; these include base32, base64, ASCII85, Uniform Resource Locator (URL) encoding, and Punycode.

Base32 and base64, which are representative binary-text encoding algorithms, converts binary data into ASCII characters, and three bytes, 24 bits, are expressed by changing them into four groups of six bits each [13]. Here, for decoding, a character for conversion is generated in units of four characters, and the character “=” is used to express an empty part. In base32 and base64, 32 and 64, respectively, refer to the number of representable characters. ASCII85, called base85, is a method of expressing five ASCII characters as four bytes of binary data, and it provides a relatively efficient encoding result compared to base64 [14]. URL encoding, called percent encoding, is an encoding method used to represent characters in URLs. In percent encoding, characters such as “!”. “*”, “@”, and “&” used in a grammatical meaning in URLs are set as reserved characters. In this case, the reserved character does not have a special grammatical meaning and it is encoded only when used as a separate character. Punycode is a method used to encode a character string composed of Unicode with only the characters allowed by the host name, which is the name of the device connected to the network. In this case, the host name is generally created as a name that is readable by humans to replace items that are less readable by humans, such as an IP address or a MAC address [15].

### 2.2. Related Works

This section describes the latest entropy-based ransomware detection research trend for detecting ransomware and the method of neutralizing it by applying the base64 encoding method, which is a previous study considered in this work.

#### 2.2.1. Ransomware Detection Methods Based on Entropy Measurement

In general, ransomware maliciously encrypts a victim’s files and blocks access to them. In the case of the Microsoft Windows operating system, data or files are encrypted using cryptography algorithms such as AES-128 or AES-256 provided by a cryptography API called Wincrypt. Therefore, most ransomware also calls these APIs to encrypt files for program stability [16]. At this time, for encryption, the ciphertext—which is the result of the encryption—has a characteristic of cryptography algorithms that generates all possible data values to be uniform. In other words, the ciphertext is generated so that specific data are not biased, because the plaintext cannot be inferred by using the value of specific data in the ciphertext. Eventually, due to the characteristics of the cryptography algorithms, the entropy of the ciphertext is increased, and ransomware-infected files can, therefore, be detected by measuring the entropy of files [17].

In [18], a zone division-based entropy detection method was proposed to measure entropy by separately diving an area that included file information such as a file signature of file contents. In [19], to detect ransomware-infected files in the system, various file formats were configured as data sets, and the entropy plot values of the files and files containing purely random numbers were compared. The larger the correlation of the plot values, the more that the file was considered to be encrypted by ransomware. As a result of the experiment, more than 80,000 files were detected to be 99.96% infected with ransomware. In [20], entropy thresholds for each file format were derived to determine whether files were infected from ransomware by file formats, and a more precise detection method was proposed using machine learning models; specifically, these were based on machine learning models such as KNN (K-Nearest Neighbor), linear regression, ridge regression, logistic regression, decision tree, random forest, SVM (Support Vector Machine), and MLP (Multi-Layer Perception). This study aimed to detect ransomware using this method, and the result showed a detection accuracy of 100%, which is significantly higher than the detection rates of 91% to 99% obtained in existing studies. In addition, [20] derived improved detection results through a comparative analysis with each entropy measurement method using Poisson distribution, Hamming distance, and the spontaneous emission measurement method, not the information entropy used in entropy measurement-based ransomware detection technology. The authors of [10,21] pointed out that, similar to the characteristics of encryption algorithms, entropy increases while files are being compressed, thus making it difficult to classify compressed files and ciphertexts solely by measuring entropy, and false detection may therefore occur. In addition, [10] pointed out that encryption of an entire file increases entropy, so encryption of a portion of a file rather than the entire file relatively decreases entropy, thus making it difficult to detect ransomware infected files.

However, for general cases, in these methods, if binary encoding algorithms are applied to the files encrypted by the ransomware, the entropy of the encrypted files is decreased, so it is impossible to detect ransomware-infected files using entropy measurement alone.

#### 2.2.2. Base64 Encoding-Based Ransomware Detection Neutralization

As described in Section 2.1.2, the different binary-text encoding algorithms are configured and operated according to each purpose. However, all encoding methods commonly perform the step of converting a string into another string, and in this process, the data of the file are converted from binary to text. After encoding, the encoding result of the file changes the entropy value of the file. Based on this fact, [10] applied the base64 encoding algorithm to the ciphertext to decrease the entropy of the encrypted file.

According to the experimental results, the proposed method verifies that it is possible to neutralize the method of detecting ransomware-infected files based on entropy. However, in the proposed method, the entropy of the base64-encoded file is lower than the entropy of the ciphertext, but it still has an obvious difference from the entropy of the plaintext. From the attacker’s point of view, this is a clue that the defender can determine that the encoding method has been applied to neutralize the ransomware detection technology; this means that the detection technology cannot be effectively neutralized. In other words, this neutralization method also has limitations. Therefore, in this paper, we propose a neutralization method for a more sophisticated file entropy measurement-based ransomware detection technology.

## 3. Analysis of Methods to Neutralize Entropy Measurement-Based Ransomware Detection Technology

In this section, we describe the experimental environment, dataset configuration, and goals of the proposed neutralization methodology to verify the possibility of neutralizing the entropy measurement-based ransomware detection technology, and we then analyze and compare the experimental results of the existing entropy measurement-based ransomware detection method, the neutralization method, and the proposed neutralization method.

### 3.1. Neutralization Methodology

**Figure 2:**
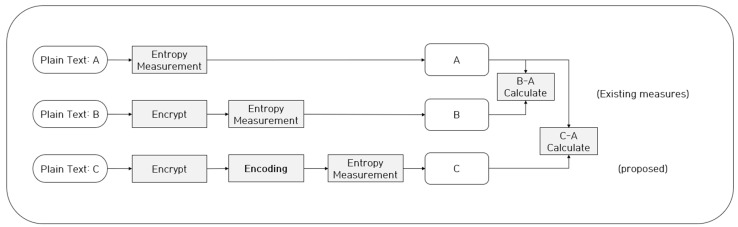
Methodology of neutralizing entropy measurement-based ransomware detection method.

As mentioned in Section 2, the existing entropy-based ransomware detection method derives a threshold by comparing the measured plaintext entropy and the entropy of the ciphertext assumed to be infected by ransomware, and the method then detects ransomware-infected files based on the derived threshold. Nevertheless, even if this method is actually infected from ransomware, there is a limitation in that it cannot detect ransomware-infected files unless the entropy is increased. Therefore, in this study, to achieve a lower entropy threshold, we used various encoding algorithms on the generated ciphertext, and Figure 2 shows the proposed neutralization method.

### 3.2. Dataset Configuration and Experiment Goals

In this study, based on the methodology described in Section 3.1, we verified the possibility of neutralizing the entropy measurement-based ransomware detection method by using the entropy of clean files not infected by ransomware, the entropy of files infected from ransomware, and the entropy of files after encoding to ransomware-infected files. Moreover, to derive optimal encoding algorithms for neutralization, we experimented with various encoding algorithms, such as base32, ASCII85, and URL encoding including base64.

For consistency, this experiment used the GovDocs1 dataset used in a previous study for entropy measurement [22]. This dataset contains a number of files, consisting of 1000 directories, each containing 1000 regular files and 1000 compressed files. This dataset also has a variety of file formats, including csv, doc, docx, dump, jpeg, log, ppt, pptx, rtf, swf, txt, xls, and xlsx. Table 1 shows the file formats and number of files used in the experiment.

To assume an environment similar to a real system infected by ransomware, this dataset includes a total of seven file formats: text files, system files, document files, image files, web page files, compressed files, and source code files. Specifically, the csv, txt, sys, dll, rtf, pdf, doc, docx, ppt, pptx, xls, xlsx, jpeg, html, zip, c, cpp, go, and py file formats are included, and among them, the file formats used in the previous study were selected as the file formats for the experiment. Here, source code files were downloaded from github sites.

In this study, we established three goals for our experimentation: The first goal was to apply the base64 encoding algorithm used in the previous study to various file formats to verify the possibility of neutralizing the ransomware detection method from the attacker’s point of view, and to draw limitations of the study by comparing and analyzing the results. The second goal was to apply various encoding algorithms including base64 and to compare and analyze the results to derive the entropy change trend for each encoding algorithm. The last goal was to analyze the entropy change trend after encoding for each file format to derive optimal encoding algorithms in which the entropy of plaintext measured for each file format and the entropy after encoding are most similar based on that derived from the second goal.

### 3.3. Limitations of Existing Studies

**Figure 3:**
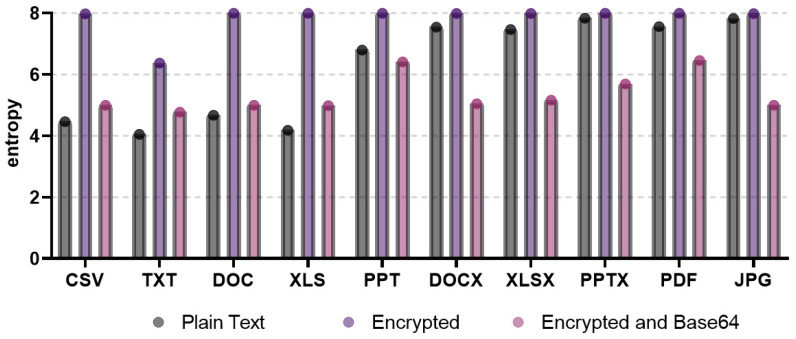
Entropy measurement results of plaintext, ciphertext, and encoded files for each file format.

In this study, we analyzed the limitations of existing studies related to the neutralization method in order to derive the optimal neutralization method from the attacker’s point of view. In [10], the base64 encoding algorithm was used to reduce the entropy involved when the ransomware encrypts the files to neutralize the entropy measurement-based ransomware detection method. To compare the entropy after encoding for each file format, Figure 3 shows the plaintext, ciphertext, and encoding results after encryption.

In the figure, the entropies of plaintext, ciphertext, and base64-encoded files are shown for various file formats, including CSV, TXT, DOC, XLS, PPT, DOCS, XLSX, PPTX, PDF, and JPEG. Altogether, the entropy measurement values of ciphertext of all file formats showed higher results than the entropy measurement values of plaintext. Hence, based on the difference in entropy between plaintext and ciphertext, ransomware-infected files are detected by deriving a threshold of the entropy. Nevertheless, the entropy measurement-based ransomware detection method does not effectively detect files infected by ransomware, because the entropy measurement results of the base64-encoded files are lower than the entropy measurement results of ciphertext, and there is no significant difference from the entropy measurement results of plaintext. In conclusion, this base64 encoding algorithm neutralizes the ransomware detection method based on entropy measurement.

On the other hand, specific file formats, such as DOCX, XLSX, PPTX, PDF, and JPG, showed similar entropy between plaintext and ciphertext. This has a limitation in that it is difficult to derive appropriate entropy thresholds. Further, the entropy of base64-encoded files is lower than the entropy of plaintext. This means that there are also limitations for defenders detecting ransomware, because the attacker encoded the file so as to bypass the detection of files infected with ransomware. As a result, from the perspective of an attacker who aims to neutralize ransomware detection, it can be determined that the attacker has applied an encoding method for neutralization, so it is not an appropriate neutralization method.

Further, specific file formats, such as CSV, TXT, DOC, and XLS, have large differences in entropy between plaintext and ciphertext. Therefore, ransomware-infected files will be detected. However, this also provides a hint for detecting ransomware-infected files, because the entropy range of base64-encoded files is located between the entropy values of plaintext and ciphertext, and the entropy of encoded files is at least slightly higher than the entropy of plaintext. Therefore, in this study, we analyzed encoding algorithms that have entropy similar to plaintext for each file format by using various encoding algorithms, including base64, to overcome the above limitations of the existing neutralization method and derive a new neutralization method with optimal performance.

### 3.4. Entropy Measurement Results by Each Encoding Algorithm

**Figure 4:**
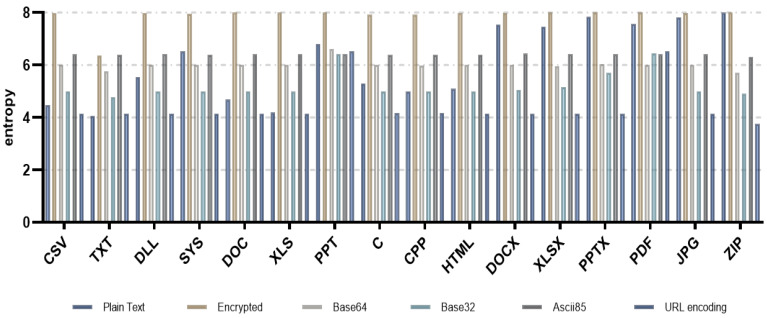
Entropy measurement results by various encoding algorithms including base32, base64, ASCII85, and URL encoding.

Figure 4 shows the entropy measurement results for each file format using various encoding algorithms including base64.

As shown in the figure, base64 has similar entropy measurement results as the previous study. For all file formats, we found that the entropy measurement values after encoding were lower than the entropy measurement values of the ciphertext. The other encoding algorithms—such as base32, ASCII85, and URL encoding—also showed that the entropy measurement values after encoding were lower than the entropy measurement values of the ciphertext for all file formats. In particular, considering the characteristic that the entropy of ransomware-infected files increases, if the entropy of the encoded file is significantly decreased and is similar to the entropy of plaintext, then it is determined to be the most suitable encoding algorithm for neutralizing the entropy measurement-based ransomware detection method.

Except for the PPT and PDF file formats, the URL encoding algorithm with the greatest decreasing entropy had the best performance, followed in descending order by base32, base64, and ASCII85. Nevertheless, as described in Section 3.3., from the ransomware attacker’s point of view, if the entropy of a file is abnormally low, it may be suspected that the ransomware detection technology or defender is attempting to neutralize the ransomware detection by an attacker; this means that the attacker fails to neutralize the ransomware detection. For this reason, the fact that the entropy of the encoded file is lower than the entropy of plaintext or ciphertext does not necessarily mean that the ransomware detection technology using entropy measurement is neutralized. It is, therefore, required to analyze the entropy measurement result of the encoded file for each file format to neutralize more sophisticated detection technology.

### 3.5. Deriving the Optimal Encoding Algorithm for Each File Format

As shown in this section, to solve the problem described above, an optimal encoding algorithm for each file format was derived by analyzing different entropy thresholds for each file format. The final goal of this study was to derive an optimal encoding algorithm neutralizing entropy measurement-based ransomware detection methods through comparison with existing studies and analyzing the entropy measurement results for each encoding algorithm. From the attacker’s point of view, if the entropy of the encoded file is almost similar to the entropy of the plaintext, then it is considered that the attacker will successfully neutralize the ransomware detection method, because the defender does not derive a threshold with which to distinguish plaintext and ciphertext from the entropy measurement result. Therefore, the optimal encoding algorithm according to each file format having these characteristics is derived using various encoding algorithms and file formats. Table 2 shows the average entropy values of plaintext, ciphertext, and encoded files for each encoding algorithm and file format as well as the entropy differences between them.

In the above table, the encoding algorithm among the base64, base32, ASCII85, and URL encoding algorithms that was most similar to the entropy of plaintext was derived. For the DLL and PPT file formats, the base64 encoding algorithm showed the most similar entropy to plaintext, and the base32 encoding algorithm showed the most similar entropy to plaintext in the DOC, HTML, C, and CPP file formats. In the SYS, DOCX, PPTX, XLSX, JPEG, and ZIP file formats, the ASCII85 encoding algorithm showed the most similar entropy to plaintext, and the URL encoding algorithm showed the most similar entropy to plaintext in the CSV, TXT, PDF, and XLS file formats. Therefore, if the best encoding algorithm derived from these file formats is applied, the ransomware detection technology can be effectively neutralized.

On the other hand, the problem of detecting files infected from ransomware was also revealed even if the encoding algorithm was applied. In the PDF file format, the base64 encoding algorithm showed the largest difference from the entropy of plaintext, and the ASCII85 encoding algorithm showed the largest difference from the entropy of plaintext in the CSV, TXT, DOC, PPT, XLS, and HTML file formats. Finally, the URL encoding algorithm showed the largest difference from the entropy of plaintext in the DLL, SYS, DOCX, PPTX, XLSX, C, CPP, JPEG, and ZIP file formats.

**Figure 5:**
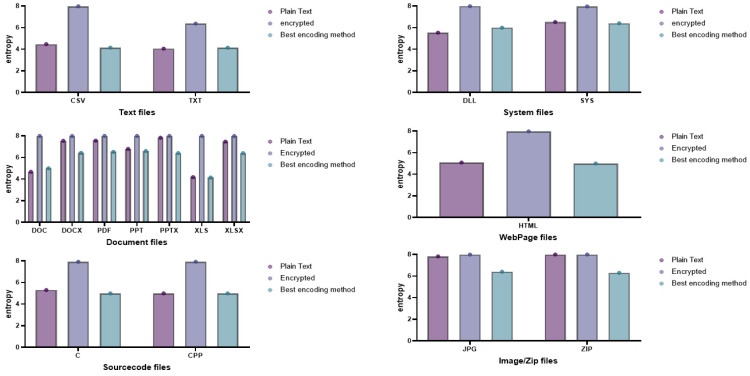
Entropy values of best encoding algorithms to bypass ransomware detection technology.

In conclusion, the base64 encoding algorithm had the best performance in a total of two file formats out of a total of 16 file formats, and the base32 encoding algorithm had the best performance in a total of four file formats. Further, the ASCII85 encoding algorithm had the best performance in a total of six file formats, and the URL encoding algorithm had the best performance in a total of four file formats. The experiment results show that the encoding algorithm that is derived to be most similar to the entropy of plaintext is different for various file formats. This means that an appropriate encoding algorithm must be applied to bypass the ransomware detection technology depending on the file format. Figure 5 shows the best encoding algorithms with the entropy values most similar to the entropy of plaintext based on the figures in Table 2.

The above figure shows the best entropy measurement results for a total of 16 file formats according to a total of seven file types: text files, system files, document files, web page files, source code files, image files, and compressed files. To neutralize the ransomware detection technology by deriving an optimal encoding algorithm that has the entropy most similar to that of plaintext, we compared the average entropy of plaintext, ciphertext, and files to which the optimal encoding algorithm is applied. Specifically, both the CSV and TXT file formats as the text file type showed that the entropy of the plaintext and the entropy of the encoded file were almost similar. Further, system files, web page files, and source code files show the same results, which means that the ransomware detection technology is neutralized by applying the best encoding algorithm proposed in this paper.

On the other hand, document files such as DOCX, PDF, PPTX, and XLSX, image files, and compressed files show similar results in the entropy of plaintext and entropy of ciphertext, and the entropy of the encoded file is significantly different from that of the plaintext. Due to compression, these file types have high entropy of plaintext. Therefore, encrypting these files instead of encoding them can neutralize ransomware detection technology.

Based on the experiment results presented in this paper, if the entropy measurement-based ransomware detection technology is neutralized by applying the optimal encoding algorithm for each file format, the neutralization performance is better than that of the neutralization method applying the base64 encoding algorithm used in previous studies.

## 4. Discussion

### 4.1. Comparison of Neutralization Accuracy with Previous Research

**Figure 6:**
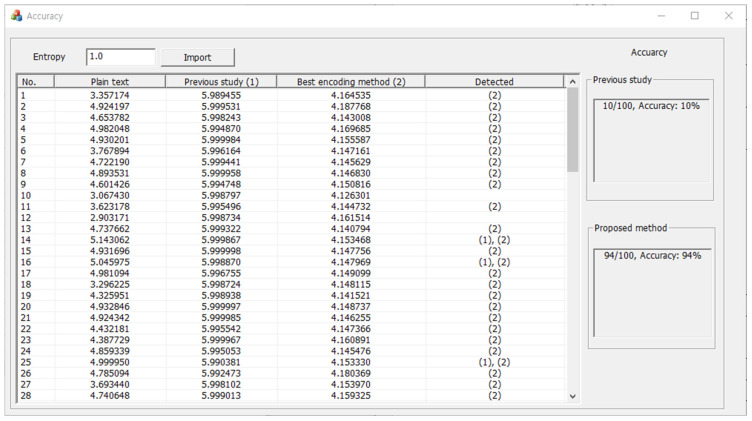
Example of a program for comparing and analyzing neutralization accuracy.

In Section 3.5, it was experimentally verified that the ransomware detection technology was more effectively neutralized by applying the optimal encoding method proposed in this paper than that used in the previous study. In this section, it is shown that, for a more objective performance comparison and analysis, the entropy threshold was set, and the neutralization accuracy of the previous study and the proposed method was derived based on the set threshold value. Based on the results shown in Table 2, the entropy threshold was set as an intermediate value between the difference between the entropy of the plaintext file and the entropy of the encoded file, and the set entropy threshold ranged from 1.0 to 2.0. Further, to derive a more accurate neutralization accuracy, the accuracy was measured while increasing the entropy threshold by 0.5. Figure 6 shows an example of a program that detects neutralization accuracy in CSV file format for comparing and analyzing neutralization accuracy.

The implemented program includes controls for inputting the entropy thresholds, the entropy of plaintext files for each file number, the entropy of files with base64 encoding methods, the entropy of files with optimal encoding methods, and columns with neutralized results. The program uses this information to measure the neutralization accuracies of the previous study and the proposed method, and the results are output. In this study, the accuracy of the neutralization method was compared and analyzed by adjusting the entropy threshold for each file format using the implemented program, and the results are shown in Table 3, Table 4 and Table 5.

When setting the entropy threshold to 1.0, the previous study and the proposed method showed respective detection rates of 10% and 94% for the CSV file format, 7% and 88% for the TXT file format, 66% for the DLL file format, 77% and 68% for the SYS file format, 24% and 48% for the DOC file format, 0% and 19% for the DOCX file format, 12% and 0% for the PDF format, 21% for the PPT file format, 3% for the PPTX file format, 18% and 69% for the XLS file format, 16% and 32% for the XLSX file format, 80% and 99% for the HTML file format, 84% and 100% for the C file format, 2% and 8% for the JPEG file format, and finally, 0% for the ZIP file format, which is a compressed file.

The same detection rates were derived for the DLL, PPT, and PPTX file formats because the optimal encoding method was base64. For all tested formats other than the SYS and PDF file formats, the neutralization accuracy of the proposed method was higher than that of the previous study. In other words, the proposed method neutralizes the entropy-based ransomware detection method more effectively.

### 4.2. Attacker’s and Defender’s Perspectives

This article describes a study that aimed to neutralize the detection of files infected with ransomware in a system. This was considered to provide an opportunity for future research to propose a defense technology for detecting and treating ransomware after a system has been infected with ransomware by detecting files infected with ransomware. Therefore, we focused on the method of neutralizing the entropy measurement-based ransomware detection method from the perspective of the attacker who produces the ransomware. Further, to derive a neutralization method with high efficiency from the attacker’s point of view, it is essential to consider the defender’s point of view. Therefore, this section describes the advantages, disadvantages, and potential attacks based on scenarios derived from the perspectives of attackers and defenders.

***Attack scenario:*** From the ransomware attacker’s point of view, since the defenders can detect files infected with ransomware by using file entropy measurement-based ransomware detection technology, there should be little or no change in the entropy of encrypted files such that they are not detected by defenders even when they are infected with ransomware. Therefore, an attacker applies various encoding methods to encrypted files so that the entropy difference from plaintext files is exceedingly small, which can cause confusion to defenders attempting to detect files infected with ransomware simply measuring the entropy of the files.

However, the file formats subject to ransomware infection are very diverse, and due to the nature of each file format, encoding may not be able to obtain an entropy value similar to the plaintext desired by the attacker. Therefore, in this study, various encoding methods were applied to various file formats to derive entropy measurement results for each file format and each encoding method, and a method for neutralizing the detection of files infected with ransomware was proposed based on the derived results.

#### 4.2.1. Attacker’s Point of View

An advantage from the attacker’s point of view is based on the entropy measurement result of the plaintext file according to each file format, and by applying various encoding methods, an optimal encoding method with a value similar to that of the plaintext file can be applied. In doing so, the entropy of plaintexts, ciphertexts, and encoded files has an obvious difference, thus overcoming the limitations of previous studies in which the neutralization method applied with encoding becomes useless. In addition, since the entropy of the encoded file and the plaintext file is almost similar, the proposed method effectively neutralizes the method of detecting files infected with entropy-based ransomware if various encoding methods are used.

However, the disadvantage is that even if certain file formats such as PDF, ZIP, DOCX, XLSX, and PPTX apply encoding methods, there is a clear difference in entropy between encoded and plaintext files, meaning it is effective to apply encryption without encoding. In addition, ransomware producers not only encrypt files, but they also have the disadvantage of requiring additional resources and time because they have to encrypt and encode files.

Despite the disadvantages mentioned above, it is difficult for the currently proposed entropy measurement-based ransomware detection technology to detect files infected with ransomware when we apply the proposed optimized encoding method, thus making it difficult to detect ransomware penetrating the system.

#### 4.2.2. Defender’s Point of View

Defenders using entropy measurement-based ransomware detection technology detect files infected with ransomware based on the entropy difference between plaintext and encrypted files. For this reason, encrypted files to which encoding has not been applied differ substantially in entropy from plaintext files, so files infected with ransomware can be effectively detected. However, if the optimal encoding method for each file format proposed in this paper is applied, there is little difference in entropy between plain text and encoded files, and it is difficult to distinguish it, thus making it virtually impossible to detect. Therefore, the defender must take countermeasures to detect files infected with ransomware targeting plaintext files and encoded files with little entropy difference.

## 5. Conclusions

This paper proposes a new effective neutralization method of the entropy measurement-based ransomware detection technology for each encoding algorithm and file format, then verifies the method based on various experimental results. The existing neutralization method bypassed the detection of files infected by ransomware by only applying the base64 encoding algorithm. However, this method only focused on decreasing the entropy of encrypted files, and it did not consider the attacker’s point of view. Consequently, attackers are required to neutralize more precisely, and it is necessary to consider an optimal encoding method with different entropy characteristics for each file format and values similar to the entropy of plaintext.

Therefore, this study derived an optimal encoding algorithm to neutralize an effective entropy measurement-based ransomware detection method through comparison with existing studies and by analyzing the entropy measurement results for each encoding algorithm. As a result, the proposed neutralization method bypassed the ransomware detection technology more effectively than the existing neutralization method. In conclusion, the results of this paper are expected to be used as baseline data for developing a method to detect ransomware-infected files more effectively.

## Figures and Tables

**Table 1:** Configuration of GovDocs1 dataset by file format and numbers of files.

File Type	File Format	Number of Files
Text file	Csv	893
	Txt	1106
System file	Sys	668
	Dll	942
Document file	Pdf	500
	Doc	500
	Docx	163
	Ppt	477
	Pptx	215
	Xls	215
	Xlsx	37
Image file	Jpeg	500
Web page file	Html	615
Compressed file	Zip	5
Source code file	C	200
	Cpp	300

**Table 2:** Entropy measurement results by each encoding algorithm and file format and entropy differences.

File Type	File Format	Plain Text	Cipher Text	BASE64 ENCODING	Base32 Encoding	ASCII85 Encoding	URL Encoding
Text file	CSV	**4.46729**	7.97813	5.99668 (−1.52939)	4.99982 (−0.53253)	**6.40287** **(−1.93558)**	**4.15058** **(+0.31672)**
	TXT	**4.05461**	6.37493	5.77204 (−1.71743)	4.77557 (−0.72096)	**6.40153** **(−2.34693)**	**4.15278** **(−0.09818)**
System file	DLL	**5.53847**	7.98340	**5.99514** **(−0.45667)**	4.99933 (+0.53914)	6.40318 (−0.86471)	**4.14982** **(+1.38865)**
	SYS	**6.51437**	7.96044	5.99482 (+0.51955)	4.99927 (+1.51510)	**6.40022** **(+0.11414)**	**4.15318** **(+2.36119)**
Document file	DOC	**4.67672**	7.99747	5.99964 (−1.32292)	**5.00007** **(−0.32335)**	**6.40776** **(−1.73104)**	4.14639 (+0.53033)
	DOCX	**7.54627**	7.98955	5.99822 (+1.54805)	5.04917 (+2.49710)	**6.43151** **(+1.11476)**	**4.14733** **(+3.39894)**
	PDF	**7.56011**	7.99737	**5.99965** **(+1.56045)**	6.45573 (+1.10438)	6.40781 (+1.15230)	**6.52028** **(+1.03983)**
	PPT	**6.79834**	7.99932	**6.60516** **(+0.19318)**	6.41370 (+0.38464)	**6.40823** **(+0.39011)**	6.51635 (+0.28199)
	PPTX	**7.83822**	7.99960	6.04482 (+1.79340)	5.69099 (+2.14723)	**6.40833** **(+1.42988)**	**4.14307** **(+3.69514)**
	XLS	**4.18588**	7.99654	5.99950 (−1.81362)	4.99000 (−0.80412)	**6.40753** **(−2.22165)**	**4.14697** **(+0.03891)**
	XLSX	**7.46922**	7.99329	5.96056 (+1.50866)	5.16354 (+2.30568)	**6.40660** **(+1.06261)**	**4.14775** **(+3.32146)**
Web page file	HTML	**5.09044**	7.97227	5.99578 (−0.90533)	**4.99936** **(+0.09109)**	**6.40147** **(−1.31103)**	4.15203 (+0.93841)
Source code file	C	**5.30585**	7.92253	5.99157 (−0.68572)	**4.99896** **(+0.30689)**	6.38732 (−1.08147)	**4.15947** **(+1.14638)**
	CPP	**4.99673**	7.92272	5.98902 (−0.99230)	**4.99882** **(−0.00209)**	6.38935 (−1.39263)	**4.16720** **(+0.82953)**
Image file	JPEG	**7.82551**	7.98869	5.99829 (+1.82722)	5.00004 (+2.82547)	**6.40548** **(+1.42003)**	**4.14940** **(+3.67611)**
Compressed file	ZIP	**7.99532**	7.99849	5.69146 (+2.30386)	4.90000 (+3.09533)	**6.30405** **(+1.69127)**	**3.74786** **(+4.24746)**

* The highlighted part in blue means the highest performance encoding method. ** The highlighted part in red means the lowest performance encoding method.

**Table 3:** Results obtained by comparing the accuracies of neutralization between the previous study and the proposed method (entropy threshold 1.0).

Entropy Threshold	File Type	File Format	Previous Study (base64 Encoding)	Proposed Method (Best Encoding Method)
1.0	Text file	CSV	10%	**94%**
		TXT	7%	**88%**
	System file	DLL	66%	
		SYS	**77%**	68%
	Document file	DOC	24%	**48%**
		DOCX	0%	**19%**
		PDF	**12%**	0%
		PPT	21%	
		PPTX	3%	
		XLS	18%	**69%**
		XLSX	16%	**32%**
	Web page file	HTML	80%	**99%**
	Source code file	C	84%	**100%**
		CPP	60%	**98%**
	Image file	JPEG	2%	8%
	Compressed file	ZIP	0%	

* The blue highlighted part means a relatively high-performance method.

**Table 4:** Results obtained by comparing the accuracies of neutralization between the previous study and the proposed method (entropy threshold 1.5).

Entropy Threshold	File Type	File Format	Previous Study (base64 Encoding)	Proposed Method (Best Encoding Method)
1.5	Text file	CSV	66%	**100%**
		TXT	33%	**98%**
	System file	DLL	75%	
		SYS	**80%**	79%
	Document file	DOC	41%	**60%**
		DOCX	28%	**75%**
		PDF	**25%**	3%
		PPT	47%	
		PPTX	3%	**28%**
		XLS	36%	**81%**
		XLSX	41%	**70%**
	Web page file	HTML	96%	**100%**
	Source code file	C	99%	**100%**
		CPP	94%	**98%**
	Image file	JPEG	9%	**34%**
	Compressed file	ZIP	0%	

* The blue highlighted part means a relatively high-performance method.

**Table 5:** Results obtained by comparing the accuracy of neutralization between the previous study and the proposed method (entropy threshold 2.0).

Entropy Threshold	File Type	File Format	Previous Study (base64 Encoding)	Proposed Method (Best Encoding Method)
2.0	Text file	CSV	81%	**100%**
		TXT	43%	**100%**
	System file	DLL	85%	
		SYS	**99%**	98%
	Document file	DOC	64%	76%
		DOCX	100%	
		PDF	**100%**	6%
		PPT	93%	
		PPTX	100%	
		XLS	61%	**93%**
		XLSX	100%	
	Web page file	HTML	99%	**100%**
	Source code file	C	100%	
		CPP	98%	**100%**
	Image file	JPEG	100%	
	Compressed file	ZIP	20%	**80%**

* The blue highlighted part means a relatively high-performance method.

## Data Availability

The data are contained within the article.

## References

[1] Everett C. (2016). Ransomware: To pay or not to pay?. Comput. Fraud Secur..

[2] (2021). KISA, Ransomware’s Latest Trend Analysis and Implications. DIGITAL & SECURITY POLICY, KISA Insight, Volume 2. https://www.kisa.or.kr/public/library/insight_View.jsp?mode=view&p_No=291&b_No=291&d_No=4&cPage=&ST=TC&SV=.

[3] Cabaj K., Gregorczyk M., Mazurczy W. (2018). Software-defined networking-based crypto ransomware detection using HTTP traffic characteristics. Comput. Electr. Eng..

[4] Paik J.-Y., Choi J.-H., Jin R., Wang J., Cho E.-S. (2018). A Storage-Level Detection Mechanism against Crypto-Ransomware. Proceedings of the Proceedings of the 2018 ACM SIGSAC Conference on Computer and Communications Security.

[5] Chen J., Wang C., Zhao Z., Chen K., Du R., Ahn G.J. (2018). Uncovering the face of android ransomware: Characterization and real-time detection. IEEE Trans. Inf. Forensics Secur..

[6] Akbanov M., Vassilakis V.G., Logothetis M.D. (2019). Ransomware detection and mitigation using software-defined networking: The case of WannaCry. Comput. Electr. Eng..

[7] Kim D., Kim S. (2015). Design of Quantification Model for Ransom Ware Prevent. WJET.

[8] Song S., Kim B., Lee S. (2016). The effective ransomware prevention technique using process monitoring on android platform. Mob. Inf. Syst..

[9] Lyda R., Hamrock J. (2007). Using entropy analysis to find encrypted and packed malware. IEEE Secur. Priv..

[10] Timothy M., Julian J., Paul W., Teo S. (2019). The inadequacy of entropy-based ransomware detection. Communications in Computer and Information Science.

[11] Lin J. (1991). Divergence measures based on the Shannon entropy. IEEE Trans. Inf. Theory.

[12] Vassilev A., Hall T.A. (2014). The importance of entropy to information security. Computer.

[13] Josefsson S. (2006). The Base16, Base32, and Base64 Data Encodings.

[14] Cooper I. (2009). MPI-Style Web Services: An Investigation into the Potential of Using Web Services for MPI-Style Applications. Ph.D. Thesis.

[15] Costello A. (2003). Punycode: A Bootstring Encoding of Unicode for Internationalized Domain Names in Applications (IDNA).

[16] Adamov A., Carlsson A., Surmacz T. An Analysis of LockerGoga Ransomware. Proceedings of the 2019 IEEE East-West Design & Test Symposium (EWDTS).

[17] Boura C., Canteaut A. (2018). On the Boomerang Uniformity of Cryptographic Sboxes. ToSC.

[18] Ju-Seong K., Kwak J. (2018). Accuracy Enhancement of Determining File Encryption Status through Divided Shannon Entropy. KIPS.

[19] Davies S., Macfarlane R., Buchanan W. (2021). Differential Area Analysis for Ransomware Attack Detection within Mixed File Datasets. J. Comput. Secur..

[20] Lee K., Lee S.Y., Yim K. (2019). Machine learning based file entropy analysis for ransomware detection in backup systems. IEEE Access.

[21] Weston P., Wolthusen S. (2014). Forensic entropy analysis of microsoft windows storage volumes. J. SAIEE Afr. Res. J..

[22] Garfinkel S., Farrell P., Roussev V., Dinolt G. (2009). Bringing science to digital forensics with standardized forensic corpora. Digit. Investig..

